# Diminished airway host innate response in people with cystic fibrosis who experience frequent pulmonary exacerbations

**DOI:** 10.1183/13993003.01228-2023

**Published:** 2024-02-22

**Authors:** Claire J. Houston, Aya Alkhatib, Gísli G. Einarsson, Michael M. Tunney, Clifford C. Taggart, Damian G. Downey

**Affiliations:** 1 Airway Innate Immunity Research Group, Wellcome-Wolfson Institute for Experimental Medicine, Queen's University Belfast, Belfast, UK; 2 School of Pharmacy, Queen's University Belfast, Belfast, UK; 3 Belfast Health and Social Care Trust, Belfast, UK; 4 Wellcome-Wolfson Institute for Experimental Medicine, Queen's University Belfast, Belfast, UK; 5 Joint senior authors

## Abstract

**Rationale:**

Pulmonary exacerbations are clinically impactful events that accelerate cystic fibrosis (CF) lung disease progression. The pathophysiological mechanisms underlying an increased frequency of pulmonary exacerbations have not been explored.

**Objectives:**

To compare host immune response during intravenous antibiotic treatment of pulmonary exacerbations in people with CF who have a history of frequent *versus* infrequent exacerbations.

**Methods:**

Adults with CF were recruited at onset of antibiotic treatment of a pulmonary exacerbation and were categorised as infrequent or frequent exacerbators based on their pulmonary exacerbation frequency in the previous 12 months. Clinical parameters, sputum bacterial load and sputum inflammatory markers were measured on day 0, day 5 and at the end of treatment. Shotgun proteomic analysis was performed on sputum using liquid chromatography-mass spectrometry.

**Measurements and main results:**

Many sputum proteins were differentially enriched between infrequent and frequent exacerbators (day 0 n=23 and day 5 n=31). The majority of these proteins had a higher abundance in infrequent exacerbators and were secreted innate host defence proteins with antimicrobial, antiprotease and immunomodulatory functions. Several differentially enriched proteins were validated using ELISA and Western blot including secretory leukocyte protease inhibitor (SLPI), lipocalin-1 and cystatin SA. Sputum from frequent exacerbators demonstrated potent ability to cleave exogenous recombinant SLPI in a neutrophil elastase dependent manner. Frequent exacerbators had increased sputum inflammatory markers (interleukin (IL)-1β and IL-8) and total bacterial load compared to infrequent exacerbators.

**Conclusions:**

A diminished innate host protein defence may play a role in the pathophysiological mechanisms of frequent CF pulmonary exacerbations. Frequent exacerbators may benefit from therapies targeting this dysregulated host immune response.

## Tweetable abstract

**Characterisation of the sputum proteome of people with CF who experience infrequent and frequent exacerbations reveals that a diminished innate host protein defence may play a role in the pathophysiological mechanisms of CF frequent exacerbations** https://bit.ly/3v7DWvr

## Introduction

Cystic fibrosis (CF) pulmonary exacerbations are important clinical events for people with CF, that alter the natural course of lung disease. They directly contribute to deterioration of lung function and are associated with accelerated lung disease progression, reduced quality of life and increased mortality risk [1–3]. While broadly recognised as periods of worsening signs and symptoms of respiratory health, manifestations of pulmonary exacerbations are highly heterogeneous [4, 5]. Despite this, treatment of pulmonary exacerbation is relatively uniform, involving administration of new antimicrobial therapy and intensification of ongoing chronic daily therapies including inhaled mucolytics, nutritional supplementation and airway clearance therapy [6]. However, the degree of treatment success is variable, as failure to recover baseline forced expiratory volume in 1 s (FEV_1_) in the short-term is observed in ≥25% of pulmonary exacerbation events and is associated with a shorter time until next pulmonary exacerbation and more frequent pulmonary exacerbations in the subsequent 3 years [7, 8]. Frequency of pulmonary exacerbations varies considerably between people with CF, and important outcomes of CF clinical trials have included a reduction in the frequency of pulmonary exacerbations and shortening the time until first pulmonary exacerbation [9–11]. In a study of CF Foundation Patient Registry data, it was demonstrated that having a history of frequent intravenous-treated pulmonary exacerbations was an independent risk factor for future *i.v.*-treated pulmonary exacerbations, and risk of *i.v.*-treated pulmonary exacerbations to be poorly predicted by patient demographic or clinical factors by comparison [12]. The underlying causative factors that lead to an increased rate of pulmonary exacerbations have not been explored.

Improving pulmonary exacerbations treatment approaches and outcomes is of paramount importance, yet very little is known about the pathophysiology of pulmonary exacerbation. While inflammation and infection are both considered to play a role in pulmonary exacerbation, within the CF lung there is complex interplay between pathogens (bacterial, viral and/or fungal) and the host's commensal taxa and immune system. As such, understanding the initiating factors and mechanisms underlying these events is immensely challenging. Moreover, there is mounting evidence of substantial intra- and inter-individual heterogeneity in both host immune and microbiological responses during pulmonary exacerbation [12–18]. Different biological pathways underlying pulmonary exacerbation may contribute to the heterogeneous responses observed. In light of this, two recent studies explored whether different phenotypes of pulmonary exacerbation with different primary drivers exist*.* One study classified pulmonary exacerbation events according to viral infection status and systemic C-reactive protein (CRP) levels, and found that biomarker profiles, clinical presentation and outcomes differed between pulmonary exacerbation classes [19]. Another study identified three clusters of pulmonary exacerbations with distinct systemic inflammatory profiles and clinical characteristics [20]. Examining the biological basis for the heterogeneity in pulmonary exacerbation responses and expanding work on pulmonary exacerbation phenotypes has great potential to inform new treatment approaches.

Proteomics is the characterisation of the entire protein content of a biological system and is a powerful tool that can be used to elucidate the underlying pathological mechanisms of disease [21]. Studies of the proteome of CF airway epithelial cells, bronchoalveolar lavage (BAL) and sputum have provided valuable insight into CF pathophysiology [22–24]. To date, proteomics has not been utilised to characterise CF pulmonary exacerbation phenotypes. We hypothesised that during a pulmonary exacerbation there would be differences in the sputum proteome of people with CF who have a history of frequent pulmonary exacerbation compared to people with CF who have a history of infrequent pulmonary exacerbation, and that the presence of unique proteins would give insight into the potential mechanisms underlying these differences in exacerbation frequency.

We characterised the sputum proteome of infrequent and frequent exacerbators, and in addition, compared various clinical, inflammatory and bacteriological measures between these patient cohorts. The potential contribution of proteolytic degradation to the altered protein levels observed in frequent exacerbators was also investigated.

## Methods

Detailed and expanded methodology is included in the supplementary material.

### Study design

We conducted an observational single-centre study of adults (aged ≥18 years) with CF at the Northern Ireland Regional Adult CF Centre, Belfast City Hospital (Belfast, UK) in line with ethical approval granted by the London-Riverside Research Ethics Committee (19/LO/0811). People with CF admitted to hospital for new *i.v.* antibiotic treatment of a pulmonary exacerbation were eligible. The diagnosis of a pulmonary exacerbation was determined independently by the clinical care team. These criteria to define a pulmonary exacerbation have been used in previous studies [5, 10]. Based on these criteria to define a pulmonary exacerbation, study participants were assigned to one of two cohorts: frequent exacerbators and infrequent exacerbators. People with CF in the frequent exacerbators group must have experienced two or more pulmonary exacerbations in the previous 12 months. The remaining participants were included in the infrequent exacerbator group. One pulmonary exacerbation event was included for each participant in the study.

## Results

### Subject clinical and demographic characteristics

The baseline clinical and demographic characteristics of the infrequent exacerbator (n=14) and frequent exacerbator (n=15) study cohorts are displayed in table 1. A total of five infrequent exacerbators and four frequent exacerbators were on single or dual CF transmembrane conductance regulator (CFTR) modulator therapy that had been commenced prior to study enrolment. Use of hypertonic saline was significantly greater in frequent exacerbators. There were no significant differences in any other baseline characteristics. Chronic *Pseudomonas aeruginosa* infection was the most common chronic respiratory infection in both infrequent (57.1%, n=8) and frequent (73.3%, n=11) exacerbators.

**TABLE 1:** Baseline demographics and clinical characteristics of study participants

	**Infrequent pulmonary exacerbations**	**Frequent pulmonary exacerbations**	**p-value**
**Participants**	14	15	
**Age years**	36.3 (18–73)	29.3 (19–45)	0.60
**Female**	7 (50.0)	11 (76.3)	0.26
**Baseline FEV_1_ % predicted**	66.21±23.1	54.20±21.1	0.15
**CFTR genotype**			
Homozygous F508del	6 (42.9)	7 (46.7)	1.00
Heterozygous F508del	6 (42.9)	6 (40.0)	1.00
**Oral antibiotic days 1 year prior to study enrolment**	39.5±33.2	62.3±41.6	0.13
***i.v.* antibiotic courses 1 year prior to study enrolment**	0.4±0.5	3.7±2.0	**0.001**
**Days of *i.v.* treatment received during study**	11.6±4	11.5±3	0.97
**CF related-comorbidities**			
CF-related diabetes	4 (28.6)	6 (40.0)	0.70
Pancreatic insufficiency	13 (92.9)	15 (100.0)	0.48
CF-related liver disease	4 (28.6)	5 (33.3)	1.00
Low bone-mineral density	5 (35.7)	7 (46.7)	0.71
**CFTR modulation**			
Any	4 (28.6)	4 (26.7)	1.00
Ivacaftor	2 (14.3)	2 (13.3)	1.00
Ivacaftor/lumacaftor	1 (7.1)	2 (13.3)	0.60
Ivacaftor/tezacaftor	1 (7.1)	0	0.48
**Long-term medications**			
Dornase alfa	13 (92.7)	15 (100.0)	0.48
Inhaled hypertonic saline	4 (64.3)	12 (80.0)	**0.0092**
Inhaled antibiotics	8 (28.6)	12 (80.0)	0.25
Macrolide	9 (57.1)	10	0.70
**Chronic infecting organism**			
* Pseudomonas aeruginosa*	8 (51.7)	11 (73.3)	0.45
* Staphylococcus aureus*	6 (42.9)	7 (46.7)	1.00
* Stenotrophomonas maltophilia*	1 (7.1)	2 (13.3)	1.00
* Achromobacter* spp*.*	2 (14.3)	2 (13.3)	1.00
* Burkholderia multivorans*	1 (7.1)	1 (6.7)	1.00

Data are presented as n, median (range), n (%) or mean±sd, unless otherwise stated. Bold type represents statistical significance. FEV_1_: forced expiratory volume in 1 s; CFTR: cystic fibrosis transmembrane conductance regulator; CF: cystic fibrosis.

### Clinical responses during pulmonary exacerbations

**FIGURE 1:**
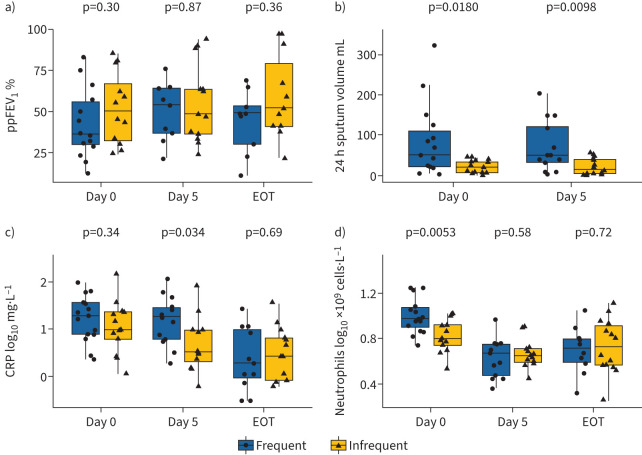
Clinical responses during pulmonary exacerbations in infrequent and frequent exacerbators. a) Percentage predicted forced expiratory volume in 1 s (ppFEV_1_); b) 24 h sputum volume; c) serum C-reactive protein (CRP) concentration; and d) serum total neutrophil count. Data are presented as median (interquartile range). EOT: end of treatment.

No significant differences in percent predicted (pp)FEV_1_ were observed between infrequent and frequent exacerbators during pulmonary exacerbation treatment (figure 1). Frequent exacerbators had a significantly increased 24-h sputum volume on days 0 and 5 of treatment compared to infrequent exacerbators. Additionally, they had significantly increased serum neutrophil counts on day 0, and CRP levels on day 5 of treatment compared to infrequent exacerbators. There was no significant difference in the mean±sd length of *i.v.* treatment between infrequent (11.6±4 days) and frequent exacerbators (11.5±3 days).

### Inflammatory and bacteriological measures during pulmonary exacerbations

**FIGURE 2:**
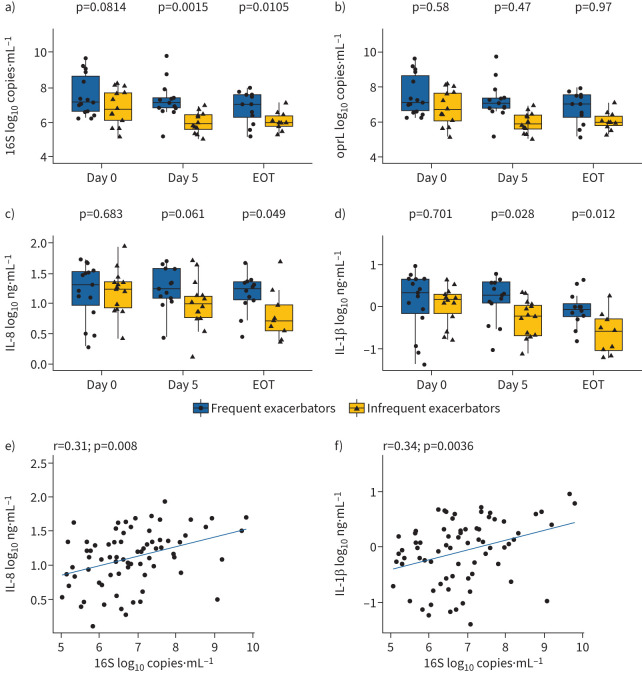
Sputum inflammatory and bacteriological measures during pulmonary exacerbation. Absolute quantification of a) total bacterial (16S) load, and b) total *Pseudomonas aeruginosa* load in sputum measured using quantitative PCR. Sputum levels of c) interleukin (IL)-8 and d) IL-1β measured using ELISA. Data are log_10_ transformed and are presented as median (interquartile range). Repeated measures correlations between e) total bacterial load and IL-8 and f) total bacterial load and IL-1β. EOT: end of treatment.

Total bacterial load was significantly greater in frequent exacerbators compared with infrequent exacerbators on day 5 and at the end of treatment (figure 2a). In contrast, *P. aeruginosa* load did not differ between these patient cohorts (figure 2b). Levels of interleukin (IL)-1β and IL-8 were not significantly different between cohorts on day 0; however, levels of IL-1β were significantly increased on day 5 and at end of treatment in frequent exacerbators compared with infrequent exacerbators, and levels of IL-8 were significantly increased at end of treatment in frequent exacerbators (figure 2c and d). Only infrequent exacerbators had a significant decline in levels of IL-1β and IL-8 during pulmonary exacerbation (supplementary table E2). Total bacterial load correlated significantly with both IL-1β and IL-8 levels (figure 2e and f).

### Differences in the sputum proteome between infrequent and frequent exacerbators

**FIGURE 3:**
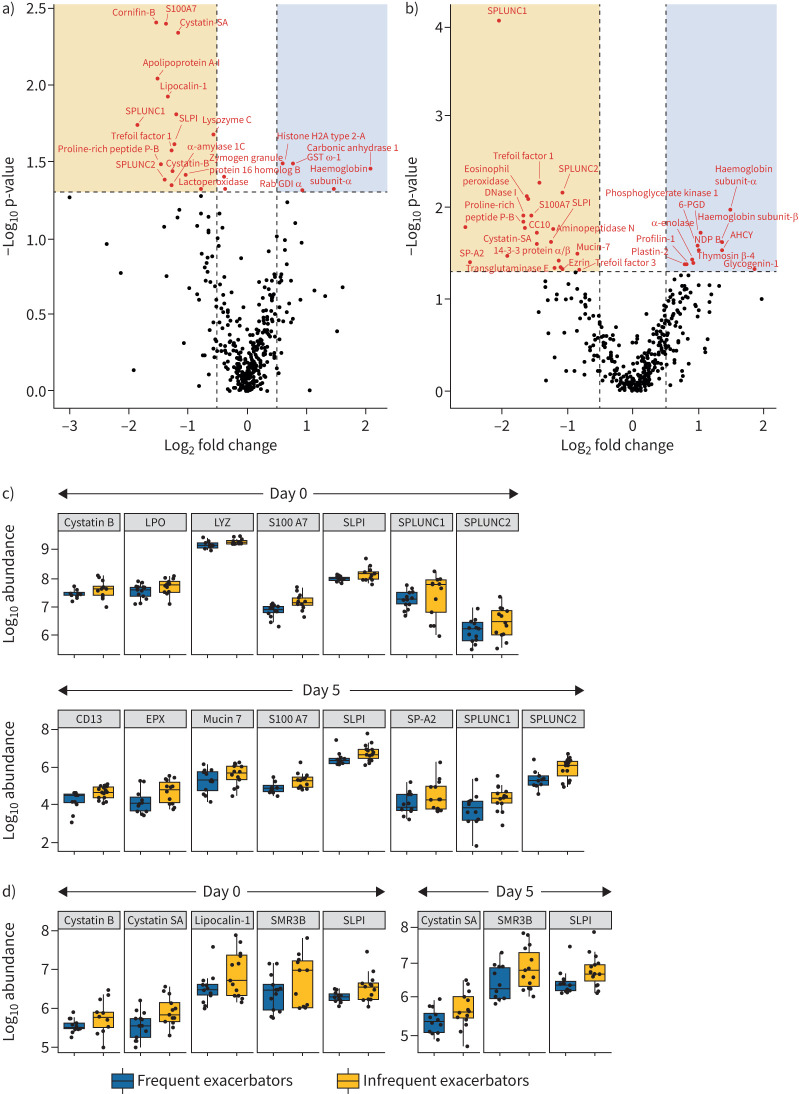
Comparison of sputum protein profiles between infrequent and frequent exacerbators during pulmonary exacerbations. Volcano plots showing differential protein abundance in sputum from infrequent exacerbators compared to frequent exacerbators on a) day 0 and b) day 5 of pulmonary exacerbations. Significantly upregulated proteins (p<0.05) are represented in red. Proteins on the outside of the vertical dashed line have a log_2_ fold change >0.5. Box plots showing log_10_ protein abundance of proteins significantly differentially enriched in sputum from infrequent exacerbators that were annotated with c) the Reactome term “innate immune function”, and d) the Gene Ontology molecular function term “protease inhibitor activity”. Data are log_10_ transformed and are presented as median (interquartile range). AHCY: adenosylhomocysteinase; CC10: club cell protein; EPX: eosinophil peroxidase; GST ω1: glutathione S-transferase ω-1; LPO: lactoperoxidase; LYZ: lysozyme C; NDP B: nucleoside diphosphate kinase B; Rab GDIα: Rab GDP dissociation inhibitor α; SLPI: secretory leukocyte protease inhibitor; SMR3B: proline-rich peptide p-B; SP-A2: surfactant protein-A2: 6-PGD: phosphogluconate dehydrogenase.

We analysed the proteome of sputum collected on day 0 and day 5 and identified 682 proteins (false discovery rate <1%), of which 360 were used for quantification (*i.e.* detected with two or more unique peptides). All proteins were common to both study cohorts. A total of 23 proteins differed in abundance on day 0 between infrequent and frequent exacerbators (figure 3a; p≤0.05). Of these proteins, 18 were higher in infrequent exacerbators and five were higher in frequent exacerbators. A total of 31 proteins differed in abundance on day 5, of which 23 proteins were higher in infrequent exacerbators and 11 proteins were higher in frequent exacerbators (figure 3b). Differentially enriched proteins are found in supplementary data file 2. The majority of proteins found at higher levels in infrequent exacerbators were secreted proteins with innate host immune functions and included proteins with antimicrobial, antiprotease and immunomodulatory activity (figure 3c and d). For example, the host defence proteins cystatin B, cystatin S, club cell protein (CC10), lipocalin-1, SPLUNC1 and secretory leukocyte protease inhibitor (SLPI) were found at higher levels in this cohort. The molecular functions, biological processes and cellular compartments associated with the proteins enriched in infrequent exacerbators are presented in table 2. Proteins found at higher levels in frequent exacerbators were associated with cellular compartments including the cytosol, exosomes and vesicles (supplementary figure E1).

**TABLE 2:** Functions of proteins enriched in infrequent exacerbator sputum

	**Term name**	**GO identifier**	**Adjusted p-value**
**Day 0**			
Molecular function	Enzyme inhibitor activity	GO:0004857	1.83×10^−05^
	Peptidase regulator activity	GO:0061134	6.22×10^−04^
Biological process	Defence response to bacterium	GO:0042742	6.37×10^−05^
	Antimicrobial humoral response	GO:0019730	5.23×10^−04^
	Negative regulation of endopeptidase activity	GO:0010951	8.26×10^−03^
Cellular compartment	Extracellular space	GO:0005615	2.67×10^−10^
	Extracellular exosome	GO:0070062	2.10×10^−05^
	Extracellular vesicle	GO:1903561	3.23×10^−05^
**Day 5**			
Molecular function	Enzyme inhibitor activity	GO:0004857	1.38×10^−02^
Biological process	Antimicrobial humoral response	GO:0019730	3.90×10^−04^
	Defence response to bacterium	GO:0042742	3.00×10^−02^
Cellular compartment	Extracellular space	GO:0005615	1.30×10^−09^
	Secretory granule	GO: 0030141	8.30×10^−05^
	Vesicle	GO:0031982	8.90×10^−05^

The proteins that were significantly higher in infrequent exacerbators were analysed using the g-profiler functional enrichment tool. The top overrepresented terms are shown and ranked by adjusted p-value. GO: gene ontology.

**FIGURE 4:**
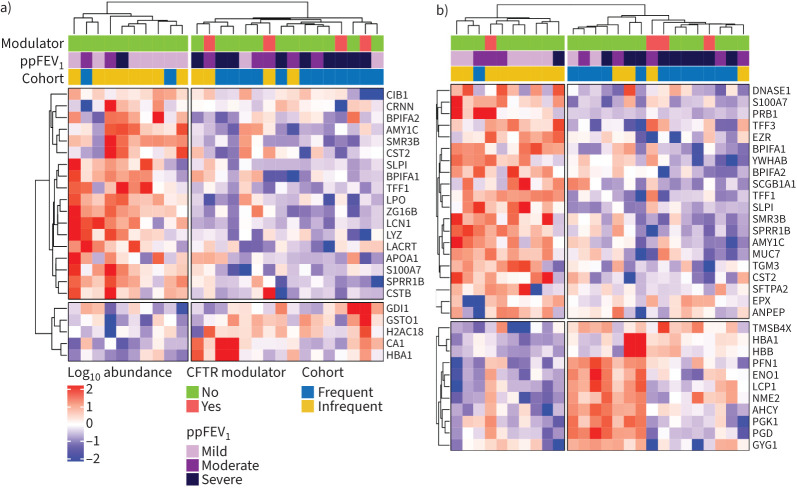
Hierarchical clustering of proteins differentially enriched between infrequent and frequent exacerbator sputum samples. Unbiased hierarchical clustering heatmaps of the log_10_ transformed and scaled abundances of significantly differentially enriched proteins (p<0.05) on a) day 0 and b) day 5 of pulmonary exacerbations. Metadata relating to each subject are shown by coloured annotations: long-term CFTR modulator use, forced expiratory volume in 1 s (FEV_1_) % predicted (mild >70%, moderate 40–70% and severe <40%) and study cohort. AHCY: adenosylhomocysteinase; AMY1C: α-amylase 1C; ANPEP: aminopeptidase N; APOA1: apolipoprotein A1; BPIFA1: BPI fold-containing family A member 1; BPIFA2: BPI fold-containing family A member 2; CA1: carbonic anhydrase 1; CIB1: calcium and integrin-binding protein 1; CRNN: cornulin; CST2: cystatin SA; CSTB: cystatin B; DNASE1: deoxyribonuclease-1; ENO1: α-enolase 1; EPX: eosinophil peroxidase; EZR: ezrin; GDI1: Rab GDP dissociation factor α; GSTO1: glutathione S-transferase omega-1; GYG1: glycogenin 1; H2AC18: histone type 2A; HBA1: haemoglobin subunit α; HBB: haemoglobin subunit β; LACRT: extracellular glycoprotein lacritin; LCN1: lipocalin-1: LCP1: plastin-2; LPO: lactoperoxidase; LYZ: lysozyme C; MUC7: mucin 7; NME2: nucleoside diphosphate kinase B; PFN1: profilin-1; PGD: 6-phosphogluconate dehydrogenase; PGK1: phosphoglycerate kinase 1; PRB1: salivary proline-rich protein 1; S100A7: S100 calcium-binding protein A7; SCGB1A1: uteroglobin; SFPTA2: surfactant protein A2; SLPI: secretory leukocyte protease inhibitor; SMR3B: submaxillary gland androgen-regulated protein 3B; SPRR1B: cornifin B: TFF1: trefoil factor 1; TGM3: transcglutaminase 3; TMSB4X: thymosin bea-4; YWHAB: 14–3–3 protein β/α; ZG16B: zymogen granule protein 16 homologue B.

Hierarchical clustering analysis was performed using the significantly differentially abundant proteins (figure 4a and b). Patient samples strongly clustered based on pulmonary exacerbations history and lung disease severity (ppFEV_1_). Samples from infrequent exacerbators that clustered with frequent exacerbators were those that had a moderate or severe ppFEV_1_.

### SLPI is only decreased in the airway of frequent exacerbators

A select number of proteins that were significantly differentially abundant in infrequent compared to frequent exacerbators were validated by Western blot and ELISA. Validation data for SPLUNC1, cystatin SA and lipocalin-1 are shown in supplementary figure E2. Several of the antiproteases that were differentially enriched during pulmonary exacerbations were found to be lower in sputum obtained 4–6 weeks post-pulmonary exacerbations treatment from a small subset of frequent exacerbators compared to infrequent exacerbators (supplementary figure E3).

**FIGURE 5:**
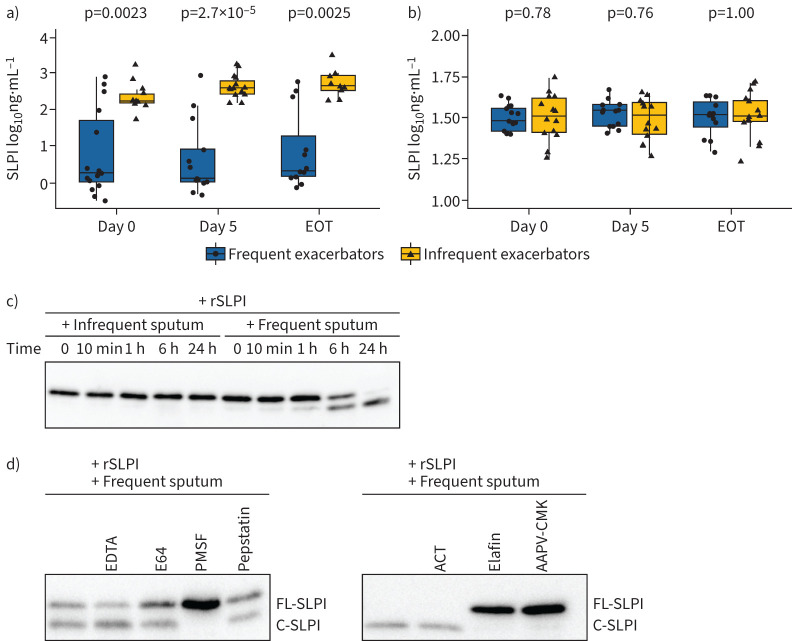
Comparison of secretory leukocyte protease inhibitor (SLPI) levels in infrequent and frequent exacerbators and degradation profile of recombinant SLPI in sputum. Comparison of SLPI levels in a) sputum and b) serum from infrequent and frequent exacerbators measured by ELISA. Data are log_10_ transformed. c) Effects of infrequent and frequent exacerbator sputum supernatant on the integrity of recombinant SLPI. d) Effects of broad-spectrum protease inhibitors, and specific serine protease inhibitors on frequent exacerbator sputum supernatant induced cleavage of SLPI. SLPI (0.166 µM) was added to pooled infrequent (n=6) or frequent (n=6) exacerbator sputum supernatant in buffer at pH 7.5 that had been pre-incubated for 1 h with protease inhibitors. Cleavage products of SLPI were assessed after 24 h at 37°C by Western blotting. EOT: end of treatment; ACT: antichymotrypsin; FL SLPI: full-length SLPI; C SLPI: cleaved SLPI.

Measurement of SLPI levels in sputum confirmed that levels were significantly lower in frequent exacerbators compared to infrequent exacerbators, not only on day 0 and day 5, but also at end of treatment (figure 5a). In view of the clear distinction in sputum SLPI levels between these patient cohorts, attention was directed toward investigating the potential mechanisms that could be responsible for the reduced levels of SLPI in frequent exacerbators. SLPI levels in serum were assessed to investigate if lower SLPI levels in frequent exacerbators was an effect compartmentalised within the airways. We observed no significant differences in serum SLPI levels between infrequent and frequent exacerbators at any time point, providing evidence that an airway-specific mechanism is responsible for reducing SLPI protein levels in frequent exacerbators (figure 5b).

### Neutrophil elastase in frequent exacerbator sputum is partly responsible for SLPI degradation

We compared the capabilities of infrequent and frequent exacerbator sputum supernatant to cleave recombinant (r)SLPI. The integrity of rSLPI after incubation at 37°C with infrequent and frequent exacerbator sputum over a 24-h time course was assessed by Western blot (figure 5c). The levels of rSLPI decreased over time when incubated in the frequent exacerbator sputum, but not when incubated in infrequent exacerbator sputum. SLPI degradation was first visible at 1 h with the appearance of a faint lower band. At 24 h, rSLPI was completely cleaved, as only a lower band was visible. These findings show that the proteolytic activity responsible for rSLPI cleavage is greater in frequent exacerbator sputum.

To identify the protease family responsible for cleaving rSLPI, we pre-incubated sputum from frequent exacerbators with or without several nonspecific protease inhibitors which target the matrix metalloproteinase, cysteine and serine protease families. After 24 h of incubation, rSLPI cleavage was only inhibited by the serine protease inhibitor PMSF, observed by the absence of the lower rSLPI band (figure 5d). Through use of the specific serine protease inhibitors we determined that neutrophil elastase was probably the protease responsible for cleaving rSLPI. Incubation of rSLPI with elafin, a specific inhibitor of neutrophil elastase and proteinase 3, as well as AAPV-CMK, a specific inhibitor of neutrophil elastase, completely inhibited cleavage of SLPI by frequent exacerbator sputum. The cathepsin G and chymase inhibitor, α-1-antichymotrypsin, failed to attenuate SLPI cleavage (figure 5d).

**FIGURE 6:**
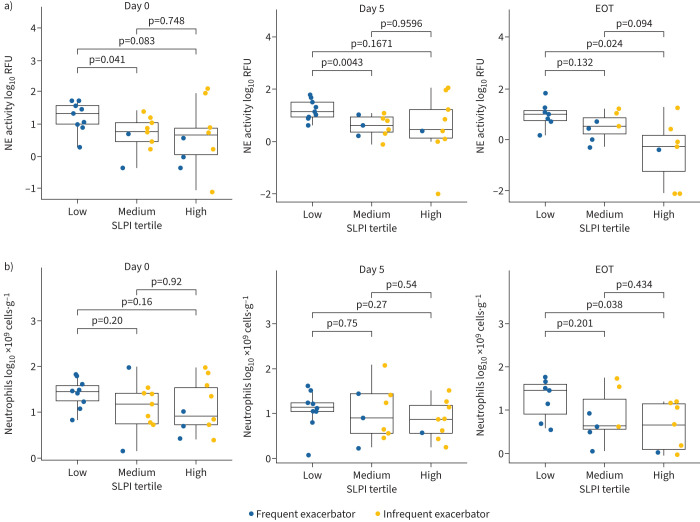
Neutrophils and neutrophil elastase (NE) activity in sputum during pulmonary exacerbations. Comparison of a) NE activity, and b) total neutrophil counts between secretory leukocyte protease inhibitor (SLPI) tertiles in sputum samples collected on day 0, day 5 and end-of-treatment (EOT) visits during pulmonary exacerbations. Values are log_10_ transformed. RFU: relative fluorescence units.

Considering these findings, neutrophil elastase enzymatic activity was then measured in individual sputum samples. We compared neutrophil elastase activity between SLPI tertiles and found that all patients in the low SLPI tertile were frequent exacerbators and had significantly higher neutrophil elastase activity than patients in the medium SLPI tertile (figure 6a). These data indicate that there is a relationship between high neutrophil elastase activity and low SLPI levels in sputum and that neutrophil elastase may be involved, either indirectly or directly, in decreasing SLPI levels. We found no significant differences in sputum total neutrophil counts between SLPI tertiles, suggesting that high levels of neutrophil elastase activity are not a result of elevated neutrophil influx into the airway and other factors may be involved (figure 6b).

### Relationship between lung disease measures and SLPI

**FIGURE 7:**
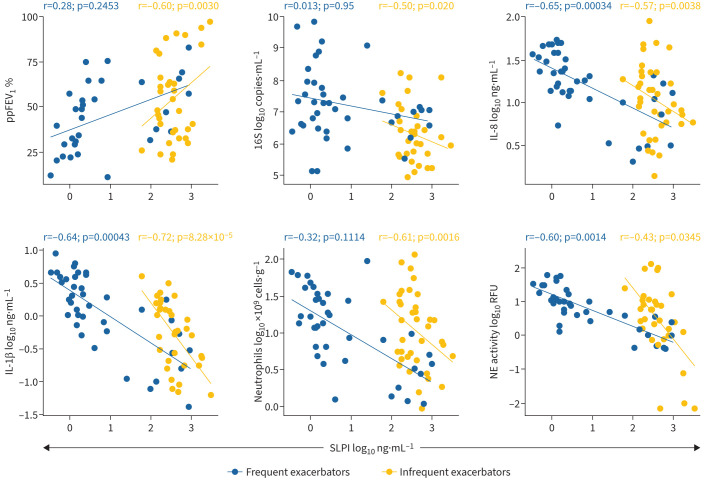
Correlations of secretory leukocyte protease inhibitor (SLPI) levels in sputum with lung function, inflammatory and bacteriological measures. Scatter plots showing sputum SLPI concentration *versus* percentage predicted forced expiratory volume in 1 s (ppFEV_1_) sputum total bacterial load, interleukin (IL)-8, IL-1β, neutrophil counts and neutrophil elastase (NE) activity. All data from day 0, day 5 and end-of-treatment sputum samples are displayed. Data are log_10_ transformed. The repeated measures correlation coefficient for each study cohort is displayed above each graph.

In both patient cohorts, significant negative correlations were found between sputum SLPI and IL-8 levels, as well as IL-1β levels and neutrophil elastase activity (figure 7). In infrequent exacerbators, SLPI had a significant positive correlation with ppFEV_1_, and a significant negative correlation with total bacterial load and total neutrophil counts (figure 7).

## Discussion

It is well established that innate immune defences are compromised in the CF airway and there is an imbalance of proteases/antiproteases, inflammation/resolution and oxidation/antioxidant states that contributes to the damaging cycle of inflammation and infection. In this study we discovered that frequent exacerbators have lower levels of specific innate immune proteins in their airways during a pulmonary exacerbation compared to infrequent exacerbators, which suggests that this may be a potential mechanism underlying a susceptibility to frequent exacerbations.

Infrequent and frequent exacerbators did not have significantly different baseline demographics and clinical characteristics, underscoring the need to characterise the biological basis of these pulmonary exacerbation phenotypes. We identified different inflammatory and bacteriological responses to *i.v.* treatment for a pulmonary exacerbation between infrequent and frequent exacerbators. Frequent exacerbators had higher levels of sputum IL-8 and IL-1β, higher systemic CRP levels and neutrophil counts, as well as a higher sputum total bacterial load. Proteomics revealed differences in the sputum proteome during pulmonary exacerbations and we found that proteins with decreased abundance in frequent exacerbators had innate host defence functions such as antiprotease, antimicrobial and immunomodulatory activities. Of these proteins, several have been studied previously and found to be dysregulated in CF and other inflammatory lung diseases including SLPI, SPLUNC1, CC10, surfactant protein A2 (SP-A2) and apolipoprotein-A1 [25–28]. Other proteins decreased in frequent exacerbators with innate host defence functions such as cystatin B, cystatin SA, lipocalin-1 and S100-A7 have not yet been studied in the context of CF lung disease and therefore may present novel immune pathways to target therapeutically. Calprotectin (S100A8/A9) is an abundant neutrophil protein in the CF lung that has been demonstrated to decrease following pulmonary exacerbations treatment; however, levels of the lesser-studied family member S100-A7 were higher in infrequent exacerbators [29]. S100-A7 plays a prominent role in the antimicrobial defence on human skin and has also been identified to be expressed by the bronchial epithelium and alveolar macrophages, indicating a potential novel and important role for this protein in airway antimicrobial defence that remains to be characterised [30].

Studies of the sputum proteome of patients with bronchiectasis have shown differences in the profile of innate host defence proteins, including some of those identified in this study, between patients with mild and severe disease. Proteins from the “neutrophil degranulation” pathway were found to be increased in patients with severe bronchiectasis, and this was linked with increased formation of neutrophil extracellular traps (NETs) [31]. Patients who tested positive for *P. aeruginosa* at pulmonary exacerbation onset also had higher levels of NET proteins, as well as lower levels of various innate host defence proteins such as SPLUNC1, cystatin S and CC10 compared to patients negative for *P. aeruginosa*. Another recent study revealed that patients with a COPD–bronchiectasis association had higher levels of neutrophil degranulation proteins and reduced levels of protease inhibitors compared to patients with COPD. The protein profiles of patients with this association more closely resembled patients with bronchiectasis [32]. The investigators proposed five endotypes to classify these patients, and, interestingly, the endotype likely to have the best prognosis was associated with increased levels of protease inhibitors and a high microbial diversity.

Protease-mediated lung injury and inflammation is a central pathophysiological mechanism of CF lung disease. Proteases can exacerbate lung injury and inflammation through multiple mechanisms such as degrading extracellular matrix proteins, enhancing the chemotactic potential of cytokines, upregulating the production of neutrophil chemoattractants and stimulating the release of damage-associated molecular patterns [33–35]. Proteases also diminish the airways innate immune capacity by degrading and inactivating their cognate antiproteases and host defence proteins in the airway surface liquid [36–38]. Reduced concentrations of SLPI, a potent neutrophil serine protease (NSP) inhibitor, and cleavage products of SLPI have been previously identified in *P. aeruginosa*-positive BAL from people with CF compared to *P. aeruginosa*-negative BAL from people with CF. This was linked to excess neutrophil elastase activity in the *P. aeruginosa*-positive BAL [25]. Interestingly, SLPI and several cysteine protease inhibitors were found to be lower in frequent exacerbators, indicating that they have a greater airway protease–antiprotease imbalance, which could exacerbate protease-mediated damage in the airway. Both the NSPs and cysteine proteases have been implicated in CF lung disease pathogenesis [39–41]. SLPI is responsible for most of the neutrophil elastase inhibitory activity in the upper respiratory tract, but has additional anti-inflammatory and antimicrobial functions, rendering it an essential protein for maintaining lung homeostasis [42, 43]. The cysteine protease inhibitors cystatin B, cystatin SA and lipocalin-1 are decreased in frequent exacerbators, but their specific role in lung disease has not been characterised. While expression of cystatin SA is limited to saliva and the nasal epithelium, it has been identified in the sputum proteome indicating it plays a role in the lung [44]. Cystatin B is a ubiquitous intracellular protein that primarily inhibits lysosomal cysteine proteases but has also been identified in body fluids [45]. In addition to its antimicrobial functions, lipocalin-1 shares three domains conserved in cystatins which are essential for their cysteine protease inhibitory activity [46]. We hypothesised that the decreased abundance of SLPI and other innate host defence proteins in frequent exacerbators was due, in part, to enhanced proteolytic degradation of these proteins. In contrast to sputum levels of SLPI which were verified by ELISA, systemic levels of SLPI did not differ between infrequent and frequent exacerbators confirming that an airway-specific mechanism was responsible for depleting SLPI. In support of our hypothesis, we found that sputum from frequent exacerbators had potent ability to cleave exogenous recombinant SLPI in a neutrophil elastase-dependent manner; in contrast, sputum from infrequent exacerbators was unable to cleave SLPI. Comparison of neutrophil elastase activity between SLPI tertiles revealed that all study participants in the lowest SLPI tertile were frequent exacerbators and had significantly higher neutrophil elastase activity compared to the other tertiles. As the increased neutrophil elastase activity in this tertile was not attributable to elevated numbers of neutrophils in the airway, it could reflect an increased sensitivity of neutrophils to activation or degranulation [47]. We also identified a strong negative relationship between SLPI levels and neutrophil elastase activity, which our findings suggest is due to direct degradation of SLPI by neutrophil elastase in the airway. It is plausible that the other innate host defence proteins decreased in frequent exacerbators are likewise decreased as a result of neutrophil elastase mediated degradation, since numerous innate immune proteins including A1AT, elafin, SPLUNC1 and SP-A have been reported previously to be susceptible to proteolytic cleavage in CF airway secretions [26, 36, 37, 48]. However, considering the complexity of protein regulation and the interplay of many host and microbial factors in the CF lung, it is possible that multiple mechanisms underlie the deficiency of innate immune proteins in frequent exacerbators. Several of these proteins including lysozyme, SLPI, SP-A and SPLUNC1 are expressed by the respiratory epithelium, particularly in the submucosal glands, and therefore may be reduced in frequent exacerbator sputum due to altered expression by these cells [22, 49]. While we did not explore the role of the microbiome, bacteria can employ various mechanisms to modulate and evade the innate immune response. For example, *P. aeruginosa* secretes LasB, which can interact with and degrade host immune proteins and inflammatory mediators [50–52].

Our findings suggest that the airway innate host protein defence is compromised to a greater extent in frequent exacerbators. The reduced abundance of these proteins could render the airway more susceptible to injury and infection, with reduced capacity to dampen and resolve destructive inflammatory responses during pulmonary exacerbations. In accordance with this, pulmonary exacerbations-associated inflammatory responses persisted despite treatment in frequent exacerbators as mean sputum IL-8 or IL-1β did not significantly decrease by the end of *i.v.*-treatment. Conversely, levels of these cytokines significantly decreased as early as day 5 of *i.v.*-treatment in infrequent exacerbators. It is possible that this is linked to a decreased abundance of protective and immunomodulatory proteins in the airway. However, pulmonary exacerbations are complex and our understanding of the precise triggers of these events is poor, with the underlying mechanisms likely to involve complex changes in the airway milieu. It is unclear if a depleted innate host defence is implicated in triggering pulmonary exacerbations events, or whether the innate defence becomes depleted as a consequence of frequent pulmonary exacerbations or other factors of CF lung disease. We found that levels of several antiproteases were lower in sputum from a small subset of frequent exacerbators at 4–6 weeks post-pulmonary exacerbations treatment. While we were unable to assess protein levels at this time point in the entire study cohort, these preliminary data indicate that these proteins may be diminished during periods of clinical stability and therefore could be chronically depleted from the airway.

A key question arising from this study relates to the impact of CFTR modulator therapy on the frequent exacerbator phenotype and whether modulators will restore the innate immune protein deficiencies? Studies have reported that initiation of elexacaftor-tezacaftor-ivacaftor (ETI) therapy leads to reductions in airway and systemic inflammation, reductions in the airway protease burden and increases in the levels of airway antiproteases including SLPI, suggesting an improvement in the protease/antiprotease imbalance [53, 54]. Furthermore, initiation of ETI shifts the CF sputum proteome closer towards the proteome of sputum from healthy controls by reducing the abundance of proteins involved in inflammatory processes such as neutrophil-mediated immunity and increasing the abundance of proteins involved in epithelial cell differentiation and protein targeting the endoplasmic reticulum [55]. However, further studies are required to determine whether these improvements are sustained beyond 12 months post-ETI and to assess the impact on lung disease progression long-term. There is also heterogeneity in treatment response, adverse drug reactions and drug interactions, a lack of access to modulators worldwide, and treatment is not available for all CF genotypes [56, 57]. Therefore, there remains an ongoing unmet need for novel therapies that modulate the innate immune response for treatment of CF lung disease. This study has highlighted that frequent exacerbators may derive benefit from therapeutics targeting the antiprotease–protease imbalance. The development of protease inhibitor therapies for CF has been greatly challenging and treatments have not progressed beyond phase II clinical trials. A major factor thought to cause difficulty in demonstrating positive therapeutic effects is CF lung disease heterogeneity; therefore, improved targeting of these drugs to the correct CF population could lead to measurable benefits [58]. As aforementioned, many of the depleted host defence proteins are epithelial-derived; therefore, the development of therapies directed at enhancing host defence protein expression by the epithelium also has promise as a novel therapeutic strategy.

Our study is limited by its single-centre design and relatively small cohort size. We were unable to include a validation cohort for proteomics due to the size of the study cohorts; however, the number of samples analysed is reasonable for the purpose of exploratory analysis and is similar to other exploratory studies [23, 59]. We validated findings for a subset of proteins by ELISA, but further validation work is required. Some of the analyses reported were not carried out for the entire study cohort due to limited sample availability or missing end-of-treatment samples and thus mean values may not be truly representative of the cohort mean. We recognise that this is a limitation to the interpretation of significant results and we seek to replicate these analyses in a larger patient cohort.

In conclusion, this study has provided novel insights into the pathophysiological mechanisms underlying frequent exacerbations which has great potential to translate into the delivery of more personalised treatment by targeting relevant disease processes.

## Supplementary material

10.1183/13993003.01228-2023.Supp1 **Please note:** supplementary material is not edited by the Editorial Office, and is uploaded as it has been supplied by the author.
Supplementary material ERJ-01228-2023.Supplement
Supplementary data file 2 ERJ-01228-2023.Data_file_2

## Shareable PDF

10.1183/13993003.01228-2023.Shareable1 This one-page PDF can be shared freely online.
Shareable PDF ERJ-01228-2023.Shareable
