# Supplementary material for: Diminished airway host innate response in people with cystic fibrosis who experience frequent pulmonary exacerbations
Source: Eur Respir J. 2024 Feb 22;63(2):2301228. doi: 10.1183/13993003.01228-2023 (PMC10882324; doi:10.1183/13993003.01228-2023)

Diminished airway host innate response in people with Cystic Fibrosis who experience frequent exacerbations

Claire J Houston[1], Aya Alkhatib[2], Gísli G Einarsson[2], Michael M Tunney[2], Clifford C Taggart[1*], Damian G Downey[3,4*]

1. Airway Innate Immunity Research Group, Wellcome-Wolfson Institute for Experimental Medicine, Queen's University Belfast, BT9 7BL, Northern Ireland, UK

2. School of Pharmacy, Queen's University Belfast, BT9 7BL, Northern Ireland, UK.

3. Belfast Health and Social Care Trust, BT9 7AB, Northern Ireland, UK

4. Wellcome-Wolfson Institute for Experimental Medicine, BT9 7BL, Queen's University Belfast, Northern Ireland, UK

* joint senior authors

Supplementary methods

Study Design

We conducted an observational single centre study of adults ($\geq$18 yr) with CF at the Northern Ireland Regional Adult CF Centre, Belfast City Hospital in line with ethical approval granted by the London-Riverside Research Ethics Committee (19/LO/0811). PwCF presenting with a PEx and admitted to hospital for intravenous (IV) antibiotic treatment were eligible. All subjects provided written informed consent. After enrolment into the study, participants were assigned to one of two groups based on their medical history in the previous 12 months: frequent exacerbators and infrequent exacerbators. PwCF in the frequent exacerbators group must have experienced at least two or more exacerbations in the previous 12 months that were treated with IV antibiotics. The remaining participants were included in the infrequent exacerbator group. There were four study visits. Visit one (D0) occurred within 24 hr of IV antibiotic treatment initiation. Visit two (D5) occurred between day 3-5 of IV treatment. Visit three (EOT) occurred within 24 h of the completion of treatment. Visit four occurred at the participants routine follow-up clinic visit at 4-6 weeks post-treatment.

Clinical data

Baseline demographics and clinical characteristics were recorded at the time of enrolment. Lung function, serum total white cell count, serum total neutrophil count, serum CRP were measured during each study visit. Twenty-four hour sputum volume was measured on D0 and D5 visits.

Sputum sample processing and differential cell staining

Spontaneously expectorated sputum samples were collected at each study visit. Two participants on D5 and seven participants at EOT were unable to spontaneously expectorate sputum. Sputum was only collected from 11 study participants at 4-6 weeks post-treatment as the remaining study participants did not have a routine post-treatment follow-up visit due to the COVID-19 pandemic. Sputum plugs were manually separated from the salivary components of sputum. Two aliquots of sputum were stored at -80°C for proteomics and

bacterial qPCR analysis. The remaining sputum was processed to obtain cell-free supernatant which was stored at -80°C. Sputum plugs were weighed and re-suspended in a volume of Dulbecco's Phosphate Buffered Saline (PBS) equal to 8 times the weight of the sputum plugs. Sputum was homogenised by repeat pipetting with a plastic transfer pipette and vortexed for 15 s. The mixture was placed on a bench rocker for 15 minutes on ice and then centrifuged at 790 x g for 10 min at 4°C. A volume of supernatant equal to four times the weight of the sputum plugs was removed and centrifuged at 1500 x g for 10 min at 4°C. The cell-free supernatant was stored at -80°C. Sputum that remained after the first centrifugation step was re-suspended in a volume of 0.2 % sputolysin (Calbiochem®, Merck) equal to 4 times the weight of sputum plugs and vortexed for 15 secs, then placed on a bench rocker for 15 mins on ice. Sputum was filtered through a 100 μM cell strainer and a total cell count performed on the filtered cell suspension by staining with trypan blue stain by counting on a haemocytometer. Differential cell counts were evaluated following cytospin preparations onto coated cytoslides (Shandon/Thermo Scientific, UK). Cells were stained with Speedy-Diff Complete Kit (CLIN-TECH LTD) and visualised using the Leica DM5500B microscope and images were captured via the Lecia AL software under X 40 objective lens. A minimum of 400 cells were counted using ImageJ software and the relative number of differential white blood cells present calculated.

Blood sample processing

Blood samples were collected at each study visit. Blood was collected in serum vacutainers and allowed to clot at room temperature for 30 mins. Samples were centrifuged at 1500 x g for 10 mins at 4°C and the cell-free supernatant was aliquoted and stored at -80°C prior to analysis.

Protein Analysis

Sputum supernatant SLPI levels were quantified by ELISA (R&D Systems) as per the manufacturer's instructions. Sputum supernatant IL-8 and IL-1β levels were quantified using ELLA Simple Plex assay (R&D systems) as per the manufacturer's instructions.

Neutrophil elastase activity assay

Neutrophil elastase (NE) activity was determined in sputum supernatant using the substrate N-Methoxysuccinyl-Ala-Ala-Pro-Val-7-amino-4-methylcoumarin (AAPV-AMC; Enzo Life Sciences, Exeter, UK) at a final concentration of 20 uM. Experiments were performed ± NE inhibitor N-Methoxysuccinyl-Ala-Ala-Pro-Val-chloromethyl ketone (AAPVCMK) at a final concentration of 1mM. The reaction buffer was 0.1 M Hepes, 0.5 M NaCl, pH 7.5. Samples were incubated in buffer ± inhibitor for 30 min at room temperature before fluorescence (substrate turnover) as determined by excitation at 360 nm and emission at 460 nm was read in a 96-well microplate reader (Synergy HT using Gen5™ software, BioTek UK). Fluorescence emission was read at 1 min intervals for 30 min at 37 °C. Results were expressed as the change in relative fluorescence units (ΔRFU) over time.

Western blotting

Western blots were performed for validation of proteomic data and for visualisation of rSLPI degradation profile. Denatured sputum supernatant samples were separated by electrophoresis on 15% SDS-polyacrylamide gels. Proteins were transferred onto a nitrocellulose membrane and probed using human anti-Cystatin S (R&D systems), human anti-Cystatin SA (R&D systems), human anti-lipocalin-1 (R&D systems), human anti-SPLUNC (R&D systems) and human anti-SLPI (Invitrogen). Binding was detected using the appropriate horseradish peroxidase-conjugated secondary antibodies, visualized by chemiluminescence (GE Healthcare UK, Buckinghamshire) and analysed using the Syngene G:Box and GeneSnap software (SynGene UK, Cambridge).

Degradation of recombinant SLPI

Recombinant SLPI (0.166 μM, R&D systems) was incubated in combination with infrequent exacerbator and frequent exacerbator sputum supernatant (pooled from N = 6 subjects on D0). Sputum was diluted 10-fold in 30 mM tris-buffered saline (TBS) pH 7.5 and incubated with rSLPI for 0 min, 10 min, 1 h, 6 h and 24 h at 37 °C. For inhibition experiments, sputum samples were pre-incubated for 1 h at 37 °C with the following protease inhibitors before incubation with rSLPI: 13 mM EDTA, 1 mM PMSF, 0.4 mM E64, 0.11 mM pepstatin A, 1μM anti-chymotrypsin (ACT), 1 μM elafin and 0.1 mM Me0Suc-AAPV-CMK. The incubation was

terminated at each time-point by boiling the sample in a heat block at 95 °C for 5 min with sample treatment buffer containing reducing agent. Samples were stored at – 20 °C until analysed by SDS-PAGE using a 15 % polyacrylamide gel, then blotted onto a nitrocellulose membrane and SLPI detected as previously described.

Sputum microbial DNA extraction

Human DNA depletion: Sputum was thawed and mixed with Sputolysin (Calbiochem®, Merck) in a 1:1 ratio and vortexed. Samples were incubated in a heat block shaker at 37 °C for 30 min at 1000 rpm then centrifuged at 8,000 x g for 5 min. The supernatant was discarded, and the pellet was resuspended in 250 µL sterile PBS and vortexed. A 200 µL volume of 5 % Saponin (Tokyo chemical industry) was added and the sample vortexed again to lyse human cells. Samples were incubated at 21 °C for 10 min. Next, 350 µL of nuclease free water was added and incubated for 30 sec then 12 µL of 5M NaCl was added to create osmotic shock. Samples were vortexed, centrifuged at 6,000 x g for 5 min and the supernatant discarded. Samples were resuspended by vortexing in 100 µL PBS and 100 µL of HL-SAN buffer (5.5 M NaCl, 100 mM $MgCl_2$, filter sterilised) and 10 µL of HL-SANDNase (250,000 units, Articzymes) added to degrade released Human DNA. After incubation at 37 °C for 15 min at 800 rpm, samples were centrifuged at 6,000 x g for 3 min and the supernatant discarded. Pellets were washed twice by vortexing in PBS (800 µL then 1 mL) to resuspend the pellet then centrifuging at 6,000 x g for 3 min. After each wash the supernatant was discarded. Pellets were resuspended in 100 µL PBS and stored at -80°C.

Microbial cell lysis: Pellets were thawed on ice and 300 µL of MagNa Pure 96 Bacterial Lysis Buffer (Roche) containing 0.35 mM lysozyme (Sigma-Aldrich) was added. Samples were vortexed and incubated for 30 min in a heat block shaker at 37 °C for 30 min at 2,000 rpm. The whole sample was transferred to 0.1 mm, 2 mL glass bead tubes (Qiagen) and homogenised in a Fast-Prep-24 rotor stator homogenizer (Mpbio) on speed 6.0 for 40 sec. Samples were incubated at 65 °C for 10 min at 1,500 rpm. A 150 µL aliquot of nuclease-free water was added to tubes before a second homogenisation in the Fast-prep homogeniser as previously described. Samples were incubated at 95 °C for 10 min at 1,000 rpm and centrifuged at 10,000 x g for 10 min at 4 °C. The supernatant was removed and stored at -80 °C for MagNA Pure DNA extraction.

MagNA Pure DNA extraction: Microbial cell lysates were thawed and DNA was extracted from 200 µL of lysate using the Roche MagNA Pure 96 automatic system according to the manufacturer's instructions. DNA amount and purity were verified using the NanoDrop spectrophotometer (Thermo Fisher Scientific). DNA samples were stored at -20°C until use.

Real-time PCR

Total 16S and *oprL* copy number in sputum was quantified by qPCR using primers and probes detailed in table 1, and validated 16S/*oprL* standards. The reaction mix comprised of 5 µL Light-cycler 480 Probes Master (Roche), 0.3 µM of each 16S primer or 0.4 µM of each *oprL* primer (Eurofins), and 0.05 µM hydrolysis probe, and was made up to a final volume of 10 µL with water. A no template control and a water-only negative control were included. Purified 16S and *oprL* standards containing 4 x 10^9 copy/µL were serially diluted 10-fold in water, creating eight standards with copies of the target amplicon ranging from 4 x 10^1 to 4 x 10^8 copy/µL. A 7.5 µL volume of reaction mix was added per well of a 96 well PCR plate (Roche) followed by 2.5 µL of standard, sample or control to give a total reaction volume of 10 µL. Each standard, sample or control was with each run in triplicate. Cycling was performed on a LightCycler 480 (Roche). Samples were initially held at 95°C for 10 min, followed by 40 cycles (16S) or 45 cycles (*oprL*) at 95°C for 10 s, 60°C for 30 s, then 72°C for 1 s. Samples were finally held at 40°C for 30 s. Standard curves with a PCR amplification efficiency of 91 – 110 % were considered acceptable.

Table E1. Bacterial qPCR probes and primers (Eurofins Genomics)

Name	Sequence	5' Modification	3' Modification
16S probe	CGT ATT ACC GCG GCT GCT GGC AC	FAM	TAM
16S-forward	TCC TAC GGG AGG CAG CAG T	-	-
16S-reverse	GGA CTA CCA GGG TAT CTA ATC CTG TT	-	-
oprL probe	AGA AGG TGG TGA TCG CAC GCA GA	FAM	BBQ650
oprL-forward	CAG GTC GGA GCT GTC GTA CTC	-	-
oprL-reverse	ACC CGA ACG CAG GCT ATG	-	-

Proteomics

A total of 53 sputum samples were analysed using an untargeted proteomic approach at the Centre for Proteome Research (CPR), University of Liverpool. Sputum samples collected on D0 (n=12 infrequent and n=14 frequent) and D5 (n=13 infrequent and n=14 frequent) of PEx were analysed, including 13 samples from the PRIVATE study (NCT0416571). Sputum samples stored at -80°C for proteomics analysis were processed and proteomics analysis was performed as previously described [1]. Briefly, samples were diluted to a final total protein amount of 25 µg in 25 mM ammonium bicarbonate. RapiGest™ SF Surfactant (Waters) was added to a final concentration of 0.05% (w/v) and incubated at 80 °C for 10 minutes. DTT was added at a final concentration of 3 mM and incubated for 10 minutes at 60 °C. Iodoacetamide was added at a final concentration of 9 mM and incubated for 30 minutes at room temperature. Mass spectrometry grade trypsin was added to samples at a protease: protein ratio of 50:1 and incubated overnight at 37 °C. Trifluoroacetic acid (TFA) was added to a final concentration of 0.5% (v/v) and incubated for 45 minutes at 37 °C with 600 rpm shaking, then samples were centrifuged for 15 minutes at 13,000 g, 4 °C. LC-MS/MS analysis was performed on the samples using the Ultimate 3000 nano system (Dionex/Thermo Fisher Scientific, Hemel Hempstead, UK) coupled to Qexactive-HF mass spectrometer (Thermo Fisher Scientific, Hemel Hempstead, UK). Peptides were loaded onto a trap column (Acclaim PepMap 100) which was set in-line with an analytical column (Easy-Spray PepMap® RSLC) (Dionex/Termo Fisher). The mass spectrometer was operated in data dependent acquisition positive (ESI+) mode and full scan MS spectra of the range 350-2000 m/z was acquired in the Orbitrap. The top 16 most intense multiply charged ions ($z \geq 2$) were sequentially isolated and fragmented in the 7octupole collision cell by high energy collisional dissociation (HCD) and detected in the Orbitrap.

Progenesis QI for Proteomics v4 (Nonlinear Dynamics) was used for label-free quantification, including run alignment, peak picking and normalisation. Database searching was carried out by the Mascot search engine (Matrix Science version 2.6.0), where samples were searched against a database containing all reviewed human sequences in the UniProt UniHumanReviewed database (updated 12/08/2020; 20,356 sequences; 11,357,197 residues). Trypsin was set as the specified enzyme, carbamidomethylation of cysteine as fixed modification, methionine oxidation as variable modification and one trypsin missed cleavage, precursor and fragment ion error tolerances were set to 10 ppm and 0.01 Da, respectively. The

false discovery rate (FDR) was calculated using the decoy database tool in MASCOT. Only those proteins identified with an FDR <1% were accepted.

Statistical analysis

Differential protein enrichment analysis of protein abundance values was performed using the SIMPLIFITM PROTIFI algorithm (https://simplifi.protifi.com/). This algorithm calculates p-values using non-parametric statistics and was performed on normalised abundances rather than logarithmic transformations. Proteins were considered significantly differentially enriched between groups if p value < 0.05 between conditions. Plotting of data and functional enrichment analysis was performed in R Studio (ver. 4.1.2). Volcano plots were created using log2 transformed fold-change values for each protein and using the R packages *ggplot2* (ver. 3.3.5), *dplyr* (ver. 1.0.8) and *ggrepel* (ver. 0.9.1). Heatmaps were created using $\log_{10}$ transformed relative abundance values of differentially enriched proteins using the R packages *ComplexHeatmap* (ver. 2.8.0) and *circlize* (ver. 0.4.13). Functional enrichment analysis of differentially enriched genes was performed using the R package *gprofiler2* (ver. 0.2.1). Gprofiler2 obtains functions from Gene Ontology (GO) and Reactome databases. Principal component analysis did not reveal any strong clustering in the overall sputum protein profiles based on study cohort. We therefore chose to focus on the group of proteins that were statistically significant which were best visualised as heatmaps and boxplots.

Plotting and statistical analysis of all other data was performed using GraphPad prism (ver. 9.3.1) or using R Studio using packages *ggplot2* (ver. 3.3.5), *ggpubr* (ver. 0.4.0), *outliers* (ver. 0.15) and *rstatix* (ver. 0.7.0). Protein immunoassay measurements, cell counts, and qPCR data were $\log_{10}$ transformed. Data are reported as mean ± SEM or median (25th-75th interquartile (IQR)) where appropriate. Normal distribution of continuous variables was assessed by D'Agostino and Pearson normality test. Means were compared by unpaired two-tailed *t*-test or two-tailed Mann-Whitney U test for non-normally distributed data. Changes between three or more study visits were analysed by paired two-way ANOVA with Tukey's multiple comparisons for non-normally distributed data. The relationship between variables was analysed using repeated measures correlation using the R package *rmcorr* (ver. 0.4.5). P < 0.05 was accepted to indicate statistical significance.

Table E2: Sputum inflammatory biomarker responses to IV-treatment. Table displaying intra-cohort changes in sputum inflammatory biomarkers between visits. Data are log_{10} transformed and are presented as mean (±SEM).

Biomarker	Cohort	D0	D5	EOT	P-value D0-D5	P-value D5-EOT	P-value D0-EOT
	Frequent	4.202 (±012)	4.26 (±0.09)	4.16 (±0.32)	0.7975	0.8648	0.3770
IL-8	Infrequent	4.20 (±0.10)	3.99 (±0.11)	3.82 (±0.14)	0.0078	0.0309	0.3446
	Frequent	3.10 (±0.17)	3.16 (±0.53)	2.95 (±0.12)	0.913	0.699	0.443
IL-1β	Infrequent	3.01 (±0.12)	2.72 (±0.12)	2.368 (±0.17)	0.023	0.019	0.098

Table E3: Sputum bacterial responses to IV-treatment. Table displaying intra-cohort changes in sputum total bacterial (16S) load and *P. aeruginosa* (oprL) load between visits. Data are log_{10} transformed and are presented as mean (±SEM).

Bacterial load (qPCR)	Cohort	D0	D5	EOT	P-value D0-D5	P-value D5-EOT	P-value D0-EOT
	Frequent	7.69 (±0.33)	7.27 (±0.31)	6.83 (±0.26)	0.3215	0.3215	0.0635
16S [log_{10} copy/mL]	Infrequent	6.85 (±0.29)	5.96 (±0.17)	5.95 (±0.14)	0.0039	0.9997	0.1198
	Frequent	6.08 (±0.53)	5.82 (±0.49)	5.38 (±0.53)	0.370	0.030	0.024
oprL [log_{10} copy/mL]	Infrequent	5.23 (±0.53)	5.23 (±0.43)	4.93 (±0.58)	0.958	0.819	0.860

References

[1] Maher RE, Barrett E, Beynon RJ, et al. The relationship between lung disease severity and the sputum proteome in cystic fibrosis. Respir Med. 2022;204:107002.

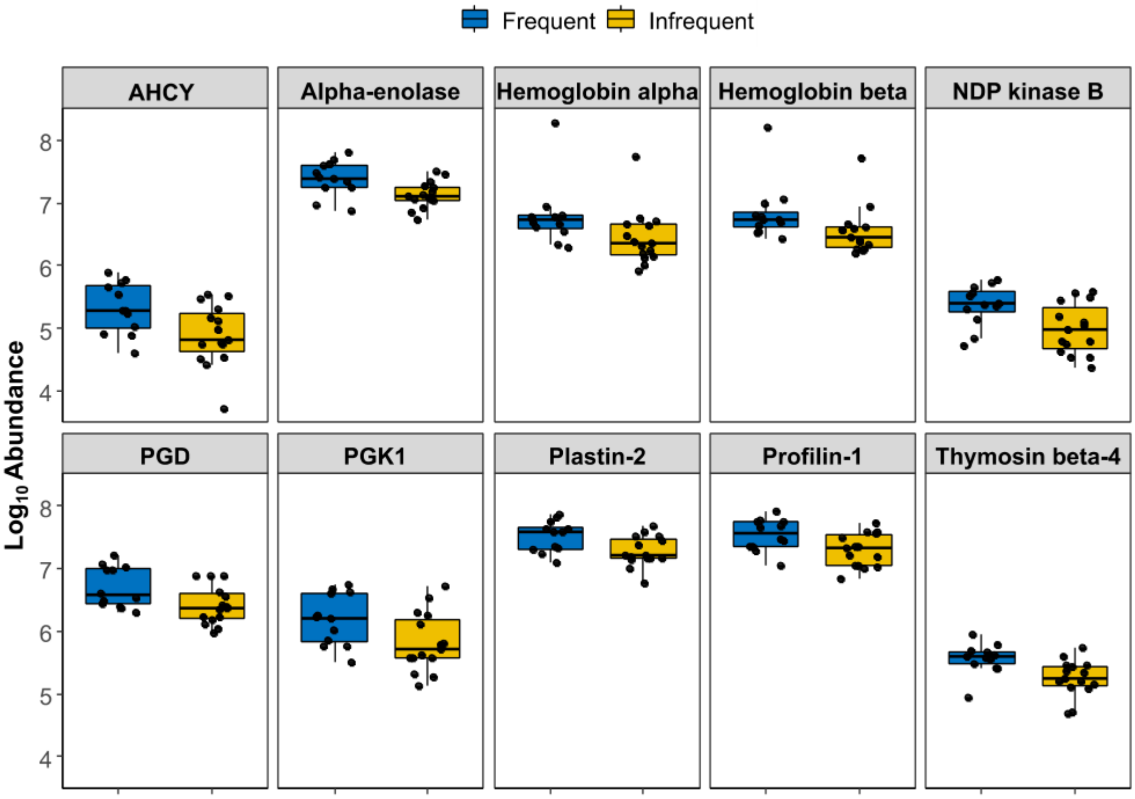

Figure E1. Cytosolic proteins enriched in frequent exacerbators. Box plots showing Log10 protein abundance of proteins significantly differentially enriched in sputum from frequent exacerbators on D5 of PEx that were annotated with the GO cellular compartment term 'cytosol'. Data are log10 transformed and are presented as median (25th-75th interquartile range).

Figure E2: Validation of proteomics results by western blot. A) Western blotting of proteins in D0 sputum supernatants from infrequent and frequent exacerbators and corresponding densitometry of B) lipocalin-1, C) cystatin SA, and D) SPLUNC1. Data are presented as mean ± SEM of n=5.

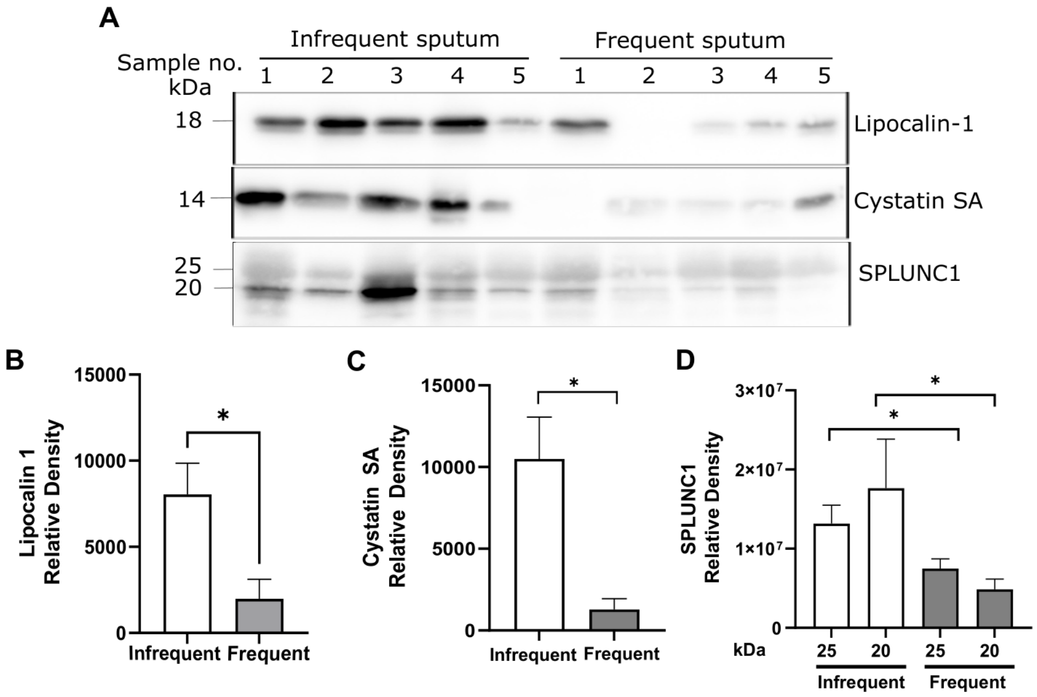

Figure E3. Antiprotease levels in sputum at 4-6 weeks post-treatment. A) Western blotting of proteins in sputum supernatants from infrequent and frequent exacerbators and B) corresponding densitometry of cystatin SA and cystatin S. C) SLPI levels determined by ELISA in sputum supernatants from infrequent and frequent exacerbators. Data are presented as mean ± SEM of n=5/6.

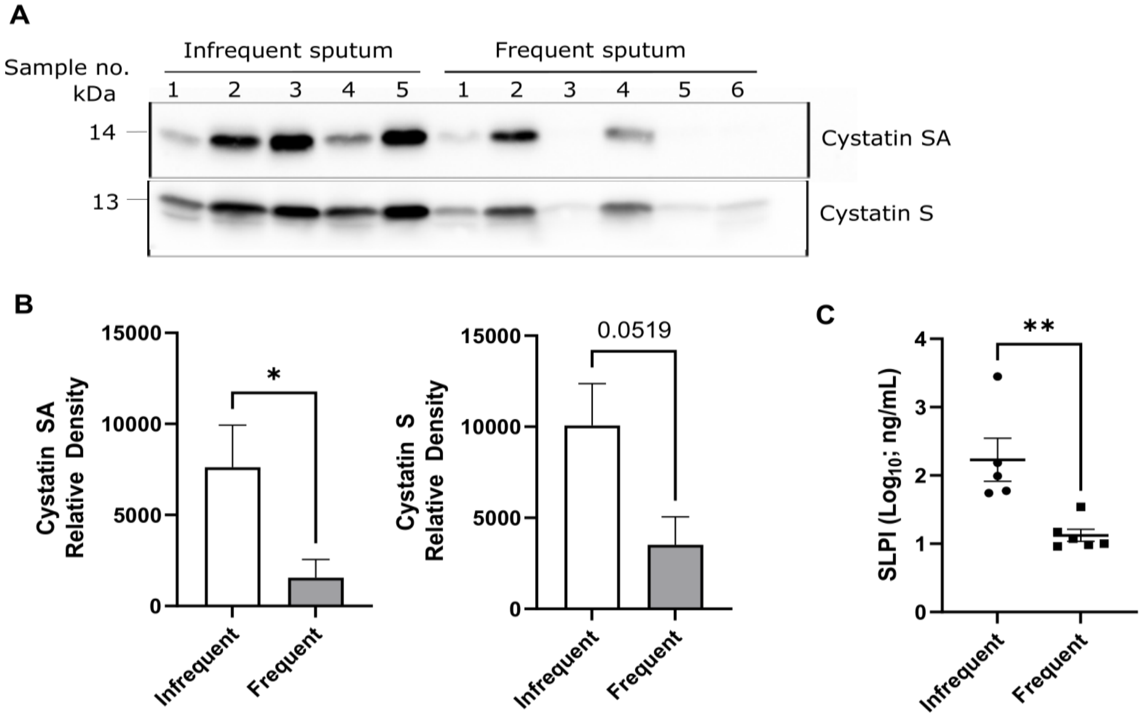

Supplement: Supplementary file 1 [file ERJ-01228-2023.Supplement.pdf]
