# Supplementary material for: Diminished airway host innate response in people with cystic fibrosis who experience frequent pulmonary exacerbations
Source: Eur Respir J. 2024 Feb 22;63(2):2301228. doi: 10.1183/13993003.01228-2023 (PMC10882324; doi:10.1183/13993003.01228-2023)
Supplement: Supplementary file 3 [file ERJ-01228-2023.Shareable.pdf]

SHAREABLE PDF

Diminished airway host innate response in people with cystic fibrosis who experience frequent pulmonary exacerbations

Claire J. Houston [1], Aya Alkhatib [2], Gísli G. Einarsson [2], Michael M. Tunney [2], Clifford C. Taggart [1,5] and Damian G. Downey [3,4,5]

[1]Airway Innate Immunity Research Group, Wellcome-Wolfson Institute for Experimental Medicine, Queen's University Belfast, Belfast, UK. [2]School of Pharmacy, Queen's University Belfast, Belfast, UK. [3]Belfast Health and Social Care Trust, Belfast, UK. [4]Wellcome-Wolfson Institute for Experimental Medicine, Queen's University Belfast, Belfast, UK. [5]Joint senior authors.

Corresponding author: Damian G. Downey (d.downey@qub.ac.uk)

Check for updates

Shareable abstract (@ERSpublications)
Characterisation of the sputum proteome of people with CF who experience infrequent and frequent exacerbations reveals that a diminished innate host protein defence may play a role in the pathophysiological mechanisms of CF frequent exacerbations https://bit.ly/3v7DWvr

Cite this article as: Houston CJ, Alkhatib A, Einarsson GG, *et al*. Diminished airway host innate response in people with cystic fibrosis who experience frequent pulmonary exacerbations. *Eur Respir J* 2024; 63: 2301228 [DOI: 10.1183/13993003.01228-2023].

This extracted version can be shared freely online.

Copyright ©The authors 2024.

This version is distributed under the terms of the Creative Commons Attribution Licence 4.0.

This article has an editorial commentary: https://doi.org/10.1183/13993003.00068-2024

Received: 20 July 2023
Accepted: 6 Dec 2023

Abstract

Rationale Pulmonary exacerbations are clinically impactful events that accelerate cystic fibrosis (CF) lung disease progression. The pathophysiological mechanisms underlying an increased frequency of pulmonary exacerbations have not been explored.

Objectives To compare host immune response during intravenous antibiotic treatment of pulmonary exacerbations in people with CF who have a history of frequent *versus* infrequent exacerbations.

Methods Adults with CF were recruited at onset of antibiotic treatment of a pulmonary exacerbation and were categorised as infrequent or frequent exacerbators based on their pulmonary exacerbation frequency in the previous 12 months. Clinical parameters, sputum bacterial load and sputum inflammatory markers were measured on day 0, day 5 and at the end of treatment. Shotgun proteomic analysis was performed on sputum using liquid chromatography-mass spectrometry.

Measurements and main results Many sputum proteins were differentially enriched between infrequent and frequent exacerbators (day 0 n=23 and day 5 n=31). The majority of these proteins had a higher abundance in infrequent exacerbators and were secreted innate host defence proteins with antimicrobial, antiprotease and immunomodulatory functions. Several differentially enriched proteins were validated using ELISA and Western blot including secretory leukocyte protease inhibitor (SLPI), lipocalin-1 and cystatin SA. Sputum from frequent exacerbators demonstrated potent ability to cleave exogenous recombinant SLPI in a neutrophil elastase dependent manner. Frequent exacerbators had increased sputum inflammatory markers (interleukin (IL)-1β and IL-8) and total bacterial load compared to infrequent exacerbators.

Conclusions A diminished innate host protein defence may play a role in the pathophysiological mechanisms of frequent CF pulmonary exacerbations. Frequent exacerbators may benefit from therapies targeting this dysregulated host immune response.
